# Sperm-related phenotypes implicated in both maintenance and breakdown of a natural species barrier in the house mouse

**DOI:** 10.1098/rspb.2012.1802

**Published:** 2012-10-10

**Authors:** Jana Albrechtová, Tomáš Albrecht, Stuart J. E. Baird, Miloš Macholán, Geir Rudolfsen, Pavel Munclinger, Priscilla K. Tucker, Jaroslav Piálek

**Affiliations:** 1 Department of Population Biology, Institute of Vertebrate Biology, Academy of Sciences of the Czech Republic, Brno and Studenec, Czech Republic; 2 Department of Zoology, Faculty of Science, Charles University in Prague, Prague, Czech Republic; 3 CIBIO, University of Porto, Campus Agrário de Vairão, Vairão, Portugal; 4 Laboratory of Mammalian Evolutionary Genetics, Institute of Animal Physiology and Genetics, ASCR, Brno, Czech Republic; 5 Department for Environmental Radioactivity, Norwegian Radiation Protection Authority, Framcenter, Tromsø, Norway; 6 Department of Ecology and Evolutionary Biology and Museum of Zoology, University of Michigan, Ann Arbor, MI, USA

**Keywords:** sperm, hybrid zone, species barrier, Y introgression, male fitness

## Abstract

The house mouse hybrid zone (HMHZ) is a species barrier thought to be maintained by a balance between dispersal and natural selection against hybrids. While the HMHZ is characterized by frequency discontinuities for some sex chromosome markers, there is an unexpected large-scale regional introgression of a Y chromosome across the barrier, in defiance of Haldane's rule. Recent work suggests that a major force maintaining the species barrier acts through sperm traits. Here, we test whether the Y chromosome penetration of the species barrier acts through sperm traits by assessing sperm characteristics of wild-caught males directly in a field laboratory set up in a Y introgression region of the HMHZ, later calculating the hybrid index of each male using 1401 diagnostic single nucleotide polymorphisms (SNPs). We found that both sperm count (SC) and sperm velocity were significantly reduced across the natural spectrum of hybrids. However, SC was more than rescued in the presence of the invading Y. Our results imply an asymmetric advantage for Y chromosome introgression consistent with the observed large-scale introgression. We suggest that selection on sperm-related traits probably explains a large component of patterns observed in the natural hybrid zone, including the Y chromosome penetration.

## 1. Introduction

Most models of speciation involve periods of hybridization [1]. Understanding the balance of forces acting during hybridization is therefore a great aid to understanding speciation itself [2]. Recent work on the European house mouse hybrid zone (HMHZ) has clarified some of the forces acting on that system. The zone of secondary contact between two house mouse subspecies, eastern *Mus musculus musculus* and western *Mus musculus domesticus*, in Europe is a model system for speciation studies. An approximately 2500 km long and 20 km wide belt of hybrids [3–10], the HMHZ is probably the most extensive and certainly one of the most intensively studied permeable species boundaries known to biology, stretching from Norway across Europe to Bulgaria [11,12]. A large array of studies carried out by multiple groups and in multiple geographical areas have shown that the narrow contact region where an explosion of admixed genotypes can be observed does not correspond to any similarly sharp ecological transition, suggesting that the dominant forces maintaining the distinction between the taxa are endogenous rather than exogenous, postmating rather than premating [3–5,7,13,14], and strong (e.g. effective selection experienced by X-linked loci being 0.23 [5]).

Multilocus studies have repeatedly shown that, while hybrids are common in the HMHZ, the spectrum of genotypes present includes few, if any, F1 or early-generation backcrosses, instead being a rich mix of late-generation backcross genotypes [5,9,10,15]. If the endogenous factors maintaining a zone are Dobzhansky–Muller incompatibilities (DMIs) [16,17], an absence of F1 and F2s in nature severely limits what laboratory crosses can tell us about the balance of forces currently maintaining the zone: DMIs come to light as combinations of alleles as yet untested by natural selection are brought together when divergent populations (A,B) meet. At contact an origin-A allele at one locus may prove incompatible with an origin-B allele at another locus (or loci). An F1 cross does not allow across-locus combinations of the incompatibility outcomes to be explored because, in an F1, an entire (haploid) source-A genome is confronted with an entire (haploid) source-B genome. An F2 cross explores across-locus combinations because recombination in F1 meiosis splices together chromosome strands of different source, allowing new combinations at *linked* DMI loci, while assortment then places these on a range of source backgrounds, allowing new combinations at *unlinked* DMI loci. Fisher [18] called the splice points between strands of different source junctions: we expect about one junction per chromosome in F2 crosses, limiting their power to explore multilocus DMI combinations, especially if members are closely linked. In a hybrid zone, the number of junctions per chromosome is a cumulant over all generations since secondary contact [19]: the process of hybridization is itself exploring the combinatorics of the DMI sets that exist between the parental taxa A,B. It is with good reason that hybrid zones are described as ‘natural laboratories for evolutionary studies’ [20]. Natural selection will favour those discovered combinations at DMI loci that are least incompatible, and we will observe their descendants. The corollary is that, when we observe a hybrid zone dominated by late-generation backcrosses (such as the HMHZ), exploring DMIs through laboratory F1 and early backcrosses is of limited relevance to the balance of forces currently acting in the zone.

Much recent progress has been made by working as closely as possible with the spectrum of hybrid genotypes actually present in nature. Vošlajerová Bímová *et al*. [21] working on wild-derived mice sampled across the hybrid zone, showed evidence supporting a degree of reinforcement selection acting on mate choice in the zone, while Baird *et al*. [22] showed, at least for the Czech/Bavarian region of the HMHZ, that hybrid mice dissected directly in the zone have reduced helminth load, eliminating these parasites as a potential force maintaining the species barrier, as had been proposed by Sage *et al*. [23] and Moulia *et al*. [24]. Similarly, previous studies have ruled out strong hybrid dysgenesis effects on some reproductive traits [25] and developmental stability [26–28].

What then are the major forces maintaining the house mouse species barrier in nature? One approach to identifying these forces is to ask under what circumstances the species barrier breaks down. In the Czech/Bavarian region of the zone, the Y chromosome of *M. m. musculus* (hereafter Y^MUS^) has penetrated the species barrier to occupy a vast area of the territory of *M. m. domesticus* (at least 330 km^2^ [6]), and a similar pattern has been found in Scandinavia also [12]. This is striking because Y chromosomes can only be carried across the zone by male mice, yet according to Haldane's rule [29], the strongest empirically supported rule of speciation (see [2] for review), the heterogametic sex should be the most affected by hybrid dysgenesis. If male hybrids are especially unfit, how then has the Y chromosome, more than any other trait studied, succeeded in crossing the species barrier? The introgression is associated with a shift in the sex ratio towards males, and Macholán *et al*. [6] suggested that this may be an example where genetic conflict has overcome the barrier to gene flow imposed by hybrid dysgenesis. An obvious place to look for phenotypes associated with genetic conflict is in the haploid phase of the mouse life cycle, where the interests of genes can be in sharp contrast to their interests in diploid individuals.

Spermatogenesis is a highly specialized process tuned to produce sperm capable of fertilization [30]. Disruption of this process at any level will negatively affect quantity and/or quality of spermatozoa. Such disruptions are observed when genetically divergent taxa are crossed in the laboratory: male F1s are often completely sterile or produce malformed sperm (reviewed in [2,29]). The relevance of these phenotypes to the balance of forces in the HMHZ has been questioned for some time [25] because of the disconnect between the early-generation hybrids studied in the laboratory versus the late-generation hybrids present in nature, but recent work [15] has considerably reduced this gap by sampling hybrids from localities within the HMHZ, crossing individuals from the same or nearby localities in the laboratory, and measuring sperm traits of their progeny. Although it does not allow for the effects of mate choice and sperm competition on the frequency spectrum of hybrids in nature, this study provides the most direct evidence to date that hybrid dysgenesis affecting sperm count (SC) and velocity may be major effects acting to maintain the mouse species barrier, while the variability of the traits indicate that these phenotypes are under highly polygenic control [15].

If the strong HMHZ species barrier is maintained by hybrid dysgenesis of sperm-related phenotypes, then the most parsimonious way to counteract that barrier effect and penetrate the zone may be to act on the same phenotypic traits. Here, we set out to test this possibility in the Czech/Bavarian region of the HMHZ where the Y^MUS^ has introgressed. Relating sperm phenotypes to HMHZ process in this region requires great care because both mate choice [21] and sperm competition are likely to influence the spectrum of hybrid genotypes present in nature. The latter effect has been demonstrated by Immler *et al*. [31], who performed a series of experiments using male sunfish caught in an area where two species (genus *Lepomis*) hybridize. They found sperm from hybrids—although able to fertilize in the absence of competition—were outcompeted by sperm of either parental species. Sperm competition is likely to be strong in the house mouse, where 12–31% of litters were shown to have been sired by multiple males [32]. As the single-male laboratory pairings chosen to produce hybrids in the study of Turner *et al*. [15] exclude the effects of both mate choice and sperm competition, an even more direct connection between genotypes assayed and genotypes present in nature is preferable for the current study.

In this paper, we describe reproductive phenotypes from 212 males live-trapped in the Czech/Bavarian HMHZ and analysed at a portable field laboratory. Specifically, we focus on SC and sperm velocity; i.e. traits commonly used as surrogates for male fertilization ability [33,34]. We evaluate how hybridization relates to these traits by estimating the hybrid index (HI) of the males using 1401 diagnostic SNPs. We furthermore investigate the sperm phenotypes in relation to the Y chromosome invasion by typing the males for a Y diagnostic marker. We discuss the sperm phenotype results in the light of both maintenance of the species barrier, and the introgression of a Y chromosome in defiance of Haldane's rule.

## 2. Material and methods

### (a) Sample collection

Sperm were obtained from 212 males live-trapped at 89 localities in the Czech/Bavarian portion of HMHZ between 2004 and 2010. After capture, mice were housed individually in clean cages with bedding material. Water and mouse-pelleted food (St1, VELAZ, Prague, Czech Republic) were available ad libitum*.* The day after capture mice were sacrificed by cervical dislocation and body length and mass were measured. The spleen was preserved in ethanol and used for DNA genotyping. Testes, epididymes and seminal vesicles were dissected and separately weighed. The contents of the *cauda epididymis*, which stores matured sperm ready to be released into the ejaculate, were used for sperm motility.

### (b) Sperm analysis

The *cauda* was cut from the right epididymis, put into 1 ml of preheated Dulbecco's Modified Eagle Medium (DMEM; Invitrogen, Carlsbad, CA) and kept at 37°C throughout the assay. It was punctured with a pair of needles and sperm were allowed to swim out for approximately 5 min. Small volumes of sperm were pipetted onto two chambers of a microscopic Leja slide (Leja, The Netherlands) which was then filmed for approximately 70 s at 10 different points at 100× magnification using a microscope (CX41, Olympus) with heating table, phase contrast and digital camera (UI-1540-C, Olympus). Sperm motility was measured in 180 males using the CEROS computer-assisted sperm analysis system (Hamilton Thorne, Inc., USA). This allows the speed of sperm to be measured in the direction of their current movement. The resulting curvilinear velocity (VCL) was used for statistical analyses as it is thought to represent motility better than simpler approximations [35].

SC was estimated from the whole left epididymis of 157 males. The organ was transferred to 2 ml of sodium citrate, cut into pieces and the solution was then homogenized with a Pasteur pipette for a few seconds. SC was assessed using a Bürker chamber following the protocol of Vyskočilová *et al*. [36] and expressed in millions per epididymis.

### (c) Genotyping

DNA from ethanol-preserved spleen was isolated using DNeasy 96 Blood & Tissue Kit (Qiagen GmbH, Hilden, Germany). Genotypes at 1401 autosomal and X-linked SNPs fixed for alternative alleles in the two subspecies were analysed using the Illumina Goldengate Assay on an Illumina Beadstation 500 (Illumina, San Diego, CA) at the University of Michigan Genotyping Core [10]. In addition, an 18-bp deletion in the Y-linked *Zfy2* gene, which is present in *M. m. domesticus* and absent in *M. m. musculus*, was analysed as described earlier [37]. The HI was quantified from the retrieved SNPs calculated for each individual as the proportion of *musculus* alleles over all SNP markers. The level of hybridization was assumed proportional to HI^2^ (see below). The effect of the Y chromosome on the measured sperm traits was estimated with reference to the state of the *Zfy2* allele. Sperm motility, SC and genotyping were analysed blindly with respect to the origin of mice.

### (d) Statistical analysis

A main focus of our inference was whether having a hybrid genotype is associated with changes in sperm phenotype. Expected heterozygosity He = 2HI(1−HI) is a straightforward measure of how hybrid an individual's genotype is. We contrasted two models for HI effects on sperm traits: the *additive* model assumes trait *T* changes linearly across the HI from *T*_1_ in one taxon to *T*_2_ in the other: *T*(HI) = (1 − HI) *T*_1_ + HI *T*_2_. The *hybrid effect* model allows for deviation *V* from this additive expectation as a function of the expected heterozygosity: *T*(HI,*V*) = *T*(HI) + *V*He. As He is quadratic in HI, the hybrid effect model can be expressed as a second-order polynomial in HI, with coefficients {*T*_1_, *T*_2_ − *T*_1_ + 2*V*, −2*V*}, while the additive model can be expressed as a first-order polynomial in HI, with coefficients {*T*_1_, *T*_2_ − *T*_1_}. Sperm velocity and mean SCs per male were approximately normally distributed (Kolmogorov–Smirnov test, *d* = 0.08, *p* > 0.10 and *d* = 0.05, *p* > 0.20, respectively), while a log transform on body mass led to a reasonable normal approximation. The polynomial nature of the models for HI effects and the approximately normal distributions of the (transformed) data allow model comparison using general linear modelling. The HI was centred on the mean value (0.497 in the analysis involving SC and 0.516 in the analysis involving VCL as dependent variable, respectively) to obtain reliable slope estimates for the linear term in the second-order polynomial [38]. All statistical analyses were performed using the statistical software R v. 2.13.1 [39]. The initial models for both the number of spermatozoa and sperm velocity included HI (with linear and quadratic terms) and the type of Y chromosome (Y^MUS^ or Y^DOM^). Body mass was added to the linear predictor as offset (i.e. coefficient for this variable not estimated [40]) in all models to control for potential effect of this trait on SC and VCL. Backward elimination of nonsignificant terms led to the selection of minimal adequate models (MAMs), i.e. models with all terms significant [41]. The significance of each MAM was evaluated by comparing the model of interest with a null model, i.e. the model containing only the overall mean [41].

### (e) Ethics statement

The study followed the experimental protocol (# 27/2007) approved by the Institutional Committee and Czech Academy of Sciences Committee for animal welfare according to Czech law.

## 3. Results

Out of all mice under study, none had the laboratory mouse F1 phenotype of zero sperm in the epididymis. In a sample of 157 males with precision SC estimates, the trait varied by two orders of magnitude from 0.38 to 29.75 × 10^6^ sperm cells per epididymis (mean 13.57 ± 5.79 [s.d.] × 10^6^). Sperm VCL was evaluated in 180 males and this trait also varied substantially from 0.00 to 159.23 µm s^−1^ (mean 100.27 ± 18.53 [s.d.] µm s^−1^).

Explaining this variation using general linear modelling, the initial full models involved linear and quadratic effects of HI (HI and HI^2^, respectively) and Y chromosome type (Y^MUS^ versus Y^DOM^), with male body mass as offset in all models (see §2d). Dropping particular terms from the models allows us to address several key questions, namely: (i) Does hybridization significantly affect SC and VCL? (ii) Are SC and VCL different in the two subspecies? (iii) Are the phenotypic traits associated with introgression of the Y^MUS^?

**Figure 1.**
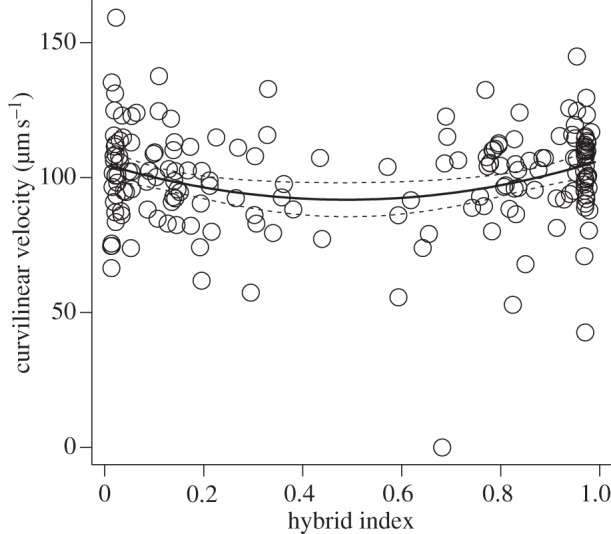
The association between HI and curvilinear sperm velocity across the HMHZ in Czech/Bavarian transect. Dashed lines represent 95% CIs.

**Figure 2.**
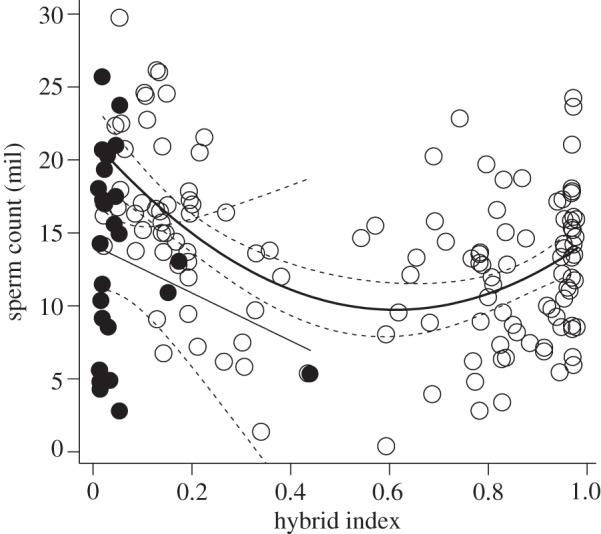
The association between HI and SC across the HMHZ in Czech/Bavarian transect**.** Open circles, individuals with Y^MUS^ chromosome; black circles, individuals with Y^DOM^ chromosome. Thick and thin solid lines display fits of data with the Y^MUS^ and Y^DOM^ chromosome, respectively. Dashed lines represent 95% CIs.

As shown in figures 1 and 2, both VCL and SC are reduced across the spectrum of hybrids relative to either parental subspecies. Removing the hybridization effect (quadratic term HI^2^) from the full models resulted in significantly poorer fits for both traits (VCL: *F* = 10.40, *Δ*d.f. = 1, *p* < 0.005; SC: *p* < 0.001 (table 1)). Thus, hybrid males have significantly lower sperm velocity and SC than the parental *M. m. musculus* and *M. m. domesticus* mice.

**Table 1.** The significance of particular terms in the best-supported models (minimal adequate models) examining the relationship between SC and sperm curvilinear velocity (VCL) with HI (linear [HI] and quadratic [HI^2^], based on 1401 SNPs) and Y chromosome type (Y) in 157 and 180 male house mice, respectively. Significance is based on type III sum of squares (controlled for effects of the remaining variables in the model). Log male body mass was included as offset in both models.

dependent variable	model term	estimate	s.e.	*F*	*Δ*d.f.	*p*
sperm count						
	HI	−6.70	1.33	11.61	1	<0.001
	HI^2^	28.58	6.44	16.27	1	<0.001
	Y	6.07	1.45	21.01	1	<0.001
sperm velocity						
	HI^2^	51.91	18.27	8.07	1	0.005

Along with a drop in male performance in response to hybridization, the results also indicated differences between subspecies in SC: elimination of the linear term (HI) from the full model led to a significant reduction in model explanatory power (*p* < 0.001, table 1). In contrast, for VCL, dropping the linear term did not lead to a significant change (*F* = 0.002, *Δ*d.f. = 1, *p* = 0.96). Thus, controlling for body size, hybridization effect and the presence of the Y chromosome type, *domesticus* males have higher SC than the *musculus* males (figure 2), but the subspecies have similar values of sperm velocity (figure 1).

Given the massive introgression of the Y^MUS^ into *domesticus* territory, performance of males carrying an introgressed Y^MUS^ is of particular interest. A Y chromosome effect was apparent in the case of SC. In *domesticus* individuals, the model predicts an increase in SC of 6.07 × 10^6^ associated with the presence of the Y^MUS^, this effect being highly significant (*p* < 0.001, table 1). Hence, *domesticus* males with introgressed Y^MUS^ have higher SC than parental *domesticus* males with a *domesticus* Y chromosome (figure 2). A different pattern was found for VCL: there was no effect of Y^MUS^ on sperm velocity (the difference between Y^MUS^ and Y^DOM^: + 6.49 µm s^−1^ ± 4.91[s.e.], *F* = 1.74, *Δ*d.f. = 1, *p* = 0.188).

In summary, the models best fitting the data for SC and VCL differed significantly in that only the former involved a linear effect of HI (difference between subspecies) and the Y chromosome effect. However, the best-supported fits for both the SC and VCL involved a quadratic effect of HI, consistent with hybrid dysgenesis. Estimates for particular parameters involved in these models are given in table 1. Both models explained a significant proportion of the variation, particularly so for SC (SC: *F*_3,153_ = 12.33, *p* < 0.001, multiple *r*^2^ = 0.195, VCL: multiple *r*^2^ = 0.044, see table 1). It should be noted that, as body size is controlled for as an offset, all the explanatory power of the models comes from just two variables: HI and Y chromosome state.

## 4. Discussion

The European HMHZ is considered a tension zone, i.e. the result of two counter-balancing processes, the immigration of individuals into the zone from either side and selection against hybrid individuals at the centre. Although the documented degree of sterility of laboratory mouse F1 males (e.g. zero sperm in the epididymis) might potentially explain the narrowness of the European HMHZ, it has repeatedly been shown that F1 males are extremely rare, if not absent entirely [5,9,10,15]. This is reconfirmed in the current study which, despite focused sampling at the hybrid zone centre (see the electronic supplementary material, figure S1), shows no individuals with HI close to ½. As noted by Turner *et al.* [15], brief consideration of the nature of the HMHZ explains this rareness of F1s: if mice disperse at the scale of 1 km^2^ per generation [5,42] and the zone is 20 km wide, then the frequency with which a pure male and a pure female both disperse across the zone in opposite directions at the same time, then meet and mate to produce an F1 offspring will be very low. If F1s are rare, then early-generation backcrosses are probably also rare. As we outlined in §1, if the zone is maintained by DMIs across loci, and dominated by late-generation backcrosses, then the relevance of early-generation laboratory backcrosses to forces currently acting in the zone is limited, because recombination and selection will tend to break up cross-DMI–loci associations and, as a consequence, the probability of co-occurrence of incompatible alleles in a hybrid may be reduced in a similar fashion to that suggested for the *Podisma* grasshopper hybrid zone [43]. There are further reasons laboratory cross results cannot be confidently extrapolated to nature: many studies have suggested that the house mouse subspecies are in the early stages of speciation, and that genes involved in sterility and reduced fertility are polymorphic within subspecies [25,36,44–46]. As the geographical distribution of these polymorphisms is largely unknown, and the geographical origin of laboratory mouse variation is poorly described, there is no guarantee that sterility genes discovered in the laboratory crosses are relevant to the tension zone process occurring in central Europe.

In this study, we have directly demonstrated a negative correlation between hybridization and sperm traits probably associated with fitness over a large sample from the spectrum of genotypes of male mice actually present in the Czech/Bavarian region of the HMHZ. We focus on SC and motility in contrast to, for example, testes size and sperm morphology through a simple decision process: when dealing with highly correlated traits, we choose that trait which is probably most closely causally linked to male reproductive success. We found that both SC and motility are significantly reduced in hybrid males compared with additive expectations. This part of our study confirms that the findings of Turner *et al*. [15] can be replicated when taking into account both natural mate choice and sperm competition, and for a different region of the HMHZ, suggesting hybrid dysgenesis of sperm traits is probably wide-spread along the course of the HMHZ.

What is the relationship between the sperm characteristics of hybrid males and their fitness? Howard *et al*. [33] argue that when, in laboratory crosses of wild-caught animals, fertile and viable offspring are produced, and such experiments consequently fail to detect a barrier to fertilization, multiple male sperm competition assays might be essential to detect prezygotic reproductive barriers. Sperm competition exerts extreme selection, as fertilization is essentially a ‘winner-takes-all’ fitness outcome. From the point of view of male fertilization success, both sperm velocity and number are judged as key determinants [47–51]. While the potential for sperm competition has been documented in *M. m. domesticus* where 12–31% of litters were shown to have been sired by multiple males [32], similar data from natural populations of *M. m. musculus* are lacking. However, competitive superiority of *M. m. musculus* sperm during *in vitro* fertilization assays [52] and higher relative testis sizes in this subspecies [15] may imply even more intense sperm competition [35,53]. Given sperm competition is widespread in the house mouse, and that sperm velocity and sperm quantity are good proxies for male competitive abilities, our results mean that hybrid males are likely to have a strong handicap in the wild, with lower fitness than their parental conspecifics. Positive correlation between SC and mean litter size sired by a male under laboratory conditions [54] suggests that the drop in SC detected in the HMHZ can lower fitness of males *per se*, and this effect can only be amplified by inter-male sperm competition.

What of hybrid males with the introgressing Y chromosome? In the section of the HMHZ we studied, the Y^MUS^ chromosome has introgressed across the zone in apparent disregard of Haldane's rule and this introgression is associated with a shift in the sex ratio in favour of males [6]. In the current study, we find that in the presence of the invading Y chromosome the most extreme reduction of SC in hybrid individuals is more than rescued, to the extent that an apparently *domesticus* male with the introgressed Y^MUS^ chromosome is expected to have higher SC than one with its consubspecific Y. This is surprising if one reasons at the population level: the combination of *domesticus* males with their own Y^DOM^ chromosome has been tested by natural selection for many generations, and so should have had an advantage on secondary contact compared with the untested combination of *domesticus* male genetic background with the Y^MUS^. However, if in the presence of the Y^MUS^ chromosome there is a sufficient sperm-related advantage, this might outweigh those disadvantages associated with moving onto a novel genetic background, allowing invasion and explaining the Czech/Bavarian observations [6]. Interestingly, a recently published study indicates a second area of introgression of Y^MUS^ into *M. m. domesticus* territory, in western Scandinavia [12]. Although the authors suggest alternative scenarios, Y introgression associated with traits increasing sperm performance cannot be disregarded as an explanation. The Scandinavian contact zone might therefore be seen as a second natural laboratory for testing the hypothesis of introgressive advantage of Y^MUS^. It seems that these may simply be the two most striking examples of a general pattern of Y introgression in the HMHZ: a survey of data from Y- and X-linked loci along a 850 km stretch of the zone strongly indicates unidirectionality in the tendency of the Y chromosome to invade [55].

A further question then arises: If Y^MUS^ have a general advantage on naturally occurring *domesticus* backgrounds, and can escape the tension zone species barrier, as in the Czech/Bavarian region, why have Y^MUS^ not invaded all of the *domesticus* range in Central Europe? The simplest explanation for the Czech/Bavarian pattern is that the escape of Y^MUS^ is relatively recent [6], but this then begs the question: Why was there a significant delay between secondary contact and escape of a supposedly universally advantageous gene? Such genes are expected to cross a tension zone with negligible delay [56,57]. One answer is that Y^MUS^ introgression requires other *musculus* factors which themselves have been trapped at the tension zone owing to tight linkage with DMIs [58]. Under tight linkage with multiple flanking DMI loci, the delay to crossing a barrier is expected to increase. The introgression of a factor on the proximal mouse X chromosome into the same geographical region as Y^MUS^ [7] despite ‘speciation genes’ in the central X chromosome [7,14] may be evidence for such a delaying mechanism, and work exploring which escaped first (Y^MUS^ or the X factor) is underway. The sex ratio distortion observed in the Y^MUS^ introgression region [6] is consistent with a conflict system dependent on sex chromosome variants, though it is unclear whether segregation distortion by Y^MUS^ is implicated. Female mice have been shown capable of altering their offspring sex ratio as a function of dietary resources [59]. Selection acting on heritable natural variation in such parental investment strategies [60,61] could be strong where new Y^MUS^-introgressed ‘supermales’ perturb an existing (female biased) balance of parental investment in the sexes [6]. Both these possibilities (Y^MUS^ segregation distortion and parental investment perturbation) allow for a limited, or even patchy (cf. [12]), spread of Y^MUS^ through *domesticus* ranges. In the case of segregation distortion, there is no reason to expect *domesticus* backgrounds to be uniform throughout their range—Y^MUS^ would be halted if they met a background against which they could not drive. Similarly, there is no reason to expect *domesticus* parental investment strategies to be uniform throughout their range, especially as evolutionarily stable strategies will depend on local resource availability [6]: it might be good to favour ‘supermales’ under one set of investment circumstances, but not under others.

Despite the numerous issues remaining to be explored, it is clear that sperm-related traits have a major effect on the HMHZ, and so a natural area for further study is to explore proximal causes for the effects we have found in the presence of the introgressing Y. As the mouse is a model organism, it is possible to use SNP-chip data to carry out genome scans for loci co-introgressing with the Y chromosome. Equally, candidate loci annotated for sperm function and traits potentially involved in genetic conflict can be examined. The very fine scale of blocks introgressing across the HMHZ suggests a very high-resolution genome scan approach might be necessary to pinpoint any genes of interest [7,62], and so both genome scan and candidate gene approaches are currently being explored. Recent analyses of Y introgression in *Drosophila* indicate genes downregulated in males with heterospecific Y chromosomes are significantly biased toward testis-specific expression patterns, the same lines showing reduced fecundity and sperm competitive ability [63]. The *Mus* Y chromosome itself has undergone recent expansion of ampliconic sequence including the spermatid-expressed gene, *Sly*, a Yq-linked regulator of post-meiotic sex chromatin, which acts to repress sex chromosome transcription in spermatids [64]. Those authors suggest *Sly* is involved in genomic conflict with one or more X-linked sex-ratio distorter genes. The conflict seems to involve postmeiotic competition between Y- and X-linked gene products that affect spermatogenesis and sex ratio [65].

Typically multilocus studies of hybrid zones show the majority of loci change coincidently and more or less concordantly in frequency gradients across the hybrid zone. Loci deviating from this pattern are generally interpreted to be the result of stochastic effects, selective advantage or traces of hybrid zone movement [7,14,21]. Here, we demonstrate in a very direct fashion the impairment of sperm performance proxies for hybrid male genotypes present in the HMHZ and further, an association of sperm production potentially linking introgression of an allele (the Y chromosome) to its reproductive performance and variation in individual fitness.
